# Sustainability Perception of Italian Consumers: Is it Possible to Replace Meat, and What Is the Best Alternative?

**DOI:** 10.3390/nu15183861

**Published:** 2023-09-05

**Authors:** Vittoria Aureli, Alessandra Nardi, Nadia Palmieri, Daniele Peluso, Jacopo Niccolò Di Veroli, Umberto Scognamiglio, Laura Rossi

**Affiliations:** 1 CREA Council for Agricultural Research and Economics—Research Centre for Food and Nutrition, 00178 Rome, Italy; vittoria.aureli@crea.gov.it (V.A.); jacoponiccolo.diveroli@crea.gov.it (J.N.D.V.); umberto.scognamiglio@crea.gov.it (U.S.); 2 Department of Mathematics, University of Rome “Tor Vergata”, 00133 Rome, Italy; alenardi@mat.uniroma2.it; 3 CREA Council for Agricultural Research and Economics—Research Centre for Engineering and Agro-Food Processing, 00015 Monterotondo, Italy; 4 Department of Biology, University of Rome “Tor Vergata”, 00133 Rome, Italy; daniele.peluso@gmail.com

**Keywords:** sustainability, consumers’ behavior, dietary recommendations, meat, meat alternative, Italy

## Abstract

Growing worldwide food demand with its environmental impacts requires a reshaping of food consumption. This study aims to evaluate the degree of Italian consumers’ awareness of sustainability and whether protein alternatives to meat could be accepted. A cross-sectional survey was carried out on a group of 815 respondents, representative of the Italian adult population for geography, gender, and age, using multivariate analysis together with cluster analysis. Lack of awareness of the consequences of food choices on the environment was found in 45% of respondents, and 51% reduced their consumption of meat. Typical foods of the Mediterranean diet (84% legumes 82% eggs, and 77% fish) were selected as the preferred sources of protein to replace meat, while insects and insect-based products were less accepted (67%). The importance of meat is the latent factor that explains more than 50% of the common variance observed in the factor analysis. The cluster analysis confirmed the importance of meat for Italian consumers, emphasizing other aspects of the sustainability of food choices. Cluster 1 (25.6%) considered meat very important. Two out of five clusters (clusters 2 and 3, 38%) considered meat replaceable in the diet, and cluster 4 (31.3%) included meat consumers that were willing to be sustainable. Cluster 5 identifies the “unsustainable consumers” (5.7%). In conclusion, besides the perceived importance of meat, there is room for recommendations for its reduction by proposing alternative foods already present in the Mediterranean diet.

## 1. Introduction

The increasing world population will inevitably cause an increase in food demand [1], especially in the most developed areas that have an unsustainable food consumption pattern, consuming more food than necessary, mainly based on animal products, processed foods, and saturated fats that inevitably have an impact on water, land use, and gas production [2]. It is estimated that 26% of anthropogenic greenhouse gas emissions (GHGE), 70% of freshwater consumption, the occupation of half the world’s habitable land, and 78% of global eutrophication of oceans and freshwater is due to agriculture [3]. Livestock production, in particular beef and lamb, contributes to 14.5% of total global GHGE [4], either in terms of GHGE or land use [5] and meat consumption largely exceeds the recommendations in several countries with an average of 34.5 kg per person per year worldwide and 80 kg per capita in Italy [6]. The food patterns of North and West Europe and the United States have the highest levels of carbon footprints, being, therefore, primarily responsible for environmental problems [7], but also bringing social, ethical, and economic implications for future generations [8].

Environmental protection is included in the framework guidelines of the World Health Organization (WHO) to be beneficial from a healthy point of view and contribute to achieving global sustainability goals [9]. In Italy too, recommendations for dietary choices that are protective of the environment and promote a sustainable food system have been included in dietary guidelines [10]. A sustainable diet is defined by the Food and Agriculture Organization (FAO) as an eating pattern with minimal environmental impact, which guarantees food security and health for future generations, which is protective and respectful of biodiversity and the environment, and is acceptable, accessible, and affordable [8]. In this sense, a diet with a low quantity of animal products and a high proportion of plant-based foods has positive effects on human cardio-metabolic health [11,12,13,14] is correlated with a reduction in overall mortality [15,16,17] and will positively impact on the environment with a relevant decrease of GHGE [18]. Cereals and legumes are characteristic vegetable protein elements of the Mediterranean dietary pattern. Legumes are an excellent source of protein that could replace the consumption of animal proteins; besides their healthy nutritional profile, legumes also represent an advantage from an environmental point of view (e.g., fixing the nitrogen in the soil, facilitating circulation of soil nutrients and water retention [19]) despite the limits of a low level of productivity in Mediterranean areas [20].

In recent years, sources of protein alternatives to meat such as algae, jellyfish, insects, and insect derivatives, traditionally used in Africa, Asia, and South America [21], have been proposed in addition to the foods traditionally recommended as meat replacements (e.g., fish, legumes, nuts, eggs, and dairy products). Despite the increasing interest in these new protein sources, people’s acceptance represents an important barrier to the consumption of very different foods in European countries [22], including Italy. Other typologies of alternative sources of proteins include lab-grown meat (from cell culture), and plant-based meat alternatives (with or without GMOs). Lab-grown meat, or in vitro meat, means meat produced through tissue engineering technologies without breeding and killing animals [23]. Laboratory production reduces the environmental impact of livestock and controls the composition and quality of meat [24]. However, cultured meat production is still very expensive [25] with the problem of consumer acceptance largely unexplored in Italy [26]. In addition, lab-grown meat needs to be assessed in terms of safety considering that it is a new product and dangers could occur from the use of specific materials, additives, ingredients (including potential allergens), and equipment used for cell-based food production [27]. Moreover, it will be necessary to understand whether the assumed benefits of the greater sustainability of lab-grown meat can be realized and guaranteed compared to conventionally produced foods [28].

At the moment, the most common meat alternatives on the market are plant-based meat substitutes, which have seen a significant increase in sales in recent years [29]. Substitutes can be found in various formats such as burgers, sausages, and ground beef, which are remarkably close to the original texture and organoleptic properties of meat [30] and have largely been accepted by consumers.

Despite the ongoing expansion of the meat alternative market, consumers are still too often unaware of the impact of their food choices on the environment. In fact, human health and animal welfare are the main motivations for consumers to reduce or even eliminate meat consumption, while environmental issues are relevant for a minority of the population [31]. According to Hartmann et al. [32], it is the lack of awareness of the negative impact that food production has on the environment that results in non-sustainable food choices.

This work originates from the idea of having a benchmark for the development of sustainability recommendations in the framework of dietary guidelines that in Italy were provided without an evaluation of consumers’ considerations of sustainability [10]. To the best of the authors’ knowledge, no similar assessments have been carried out in Italy on a representative sample of the population. Hence, the research questions and the gaps that this study intended to fill in were (i) how much attention do Italian consumers pay to the environmental consequences of their dietary choices? (ii) To what extent is meat considered essential or are alternatives acceptable to Italian consumers? (iii) What kind of policymaking would consumers welcome to increase the sustainability of their eating behaviors? (iv) Is it possible to identify socio-demographic characteristics related to the sustainability of food consumption? These data have practical applications related to the possibility of providing real-life suggestions aimed at improving the sustainability of consumer food choices.

The objectives of this study were to evaluate the degree of Italian consumers’ awareness of food sustainability and whether alternative proteins to meat could be recommended in the context of dietary guidelines and nutritional advice, hypothesizing a conservative attitude of Italian consumers toward new foods that are markedly different to traditional foods [26,33].

## 2. Materials and Methods

### 2.1. Design of the Study

A cross-sectional survey was carried out in Italy on 815 adults (over 18 years), nationally representative for geography, gender, and age. The fieldwork was conducted in the period between 22 and 28 March 2022 by a specialized market research agency, SWG Italy^®^. The data were collected through online interviews using the CAWI (Computer Assisted Web Interviewing) technique on a group of adults residing in Italy, extracted from a panel that includes over 60,000 individuals, profiled according to the main national socio-demographic variables. A random selection method to identify the respondents was used, stratifying area of residence, age group, and gender. To improve representativeness for education, a Random Iterative Weighting was used. The target distribution was the most recently available distribution of educational level in Italy (at the time of the survey), stratified according to the area of residence, age group, and gender, as provided by the National Institute of Statistics [34]. The survey size was defined to guarantee a maximum margin of error of 3.5% at 95% confidence intervals (CI). Before the start of data collection, respondents were required to sign a privacy agreement and consent form for personal data collection and processing in accordance with the Italian data protection law (Legislative Decree 101/2018), in line with the European Commission’s general data protection regulation (679/2016). Participants were informed about the objective of the research and the consequent statistical analysis. Participation in the study was fully voluntary and anonymous, and subjects could withdraw from the survey at any time and for any reason. The study was conducted according to the guidelines of the Declaration of Helsinki [35], and all procedures involving research study participants were approved and are in line with the SWG code of conduct [36]. As the assessment did not involve any invasive procedures or induce any changes in dietary patterns, the study did not require approval from the ethics committee.

### 2.2. Assessment Tool

The questionnaire used in the present paper was previously validated on the Italian population. The questionnaire resulting from the validation process can be found in the supplementary material of the paper of Aureli et al. [37]. No further modifications were carried out on the assessment tool. The questionnaire was conceived in order to assess the perception of Italian consumers on the theme of the environmental impact of food choices. The outcomes of the assessment could be used for the development of tailored recommendations.

In synthesis, a multi-section questionnaire was administered with an initial part covering socio-demographic information (gender, age, region of residence, educational level, and income) and self-reported weight and height. The key elements of the assessment tool consisted of 12 questions that comprise 71 items from which three sections could be identified: (i) food sustainability knowledge (4 questions accounting for 30 items); (ii) sources of proteins alternative to meat (3 questions accounting for 20 items); (iii) eating behaviors (5 questions accounting for 21 items). Continuous scale questions were mingled with categorical questions requiring yes/no responses. In the case of continuous scale questions, answers were provided through a 10-point Likert scale (1 corresponding to “strongly disagree” to 10 corresponding to “strongly agree”).

### 2.3. Data Analysis

Absolute frequencies and percentages were used to describe categorical items; continuous items were summarized as mean and standard deviation (SD).

In order to reduce data dimension, Factor Analysis (FA) was performed. The Principal Component Analysis (PCA) method was used for the extraction of factors. With the aim of optimizing the loading factor of each item Varimax rotation was applied. For all the extracted factors, eigenvalues, proportions, and cumulative proportions of explained common variance were computed. The proportion of explained common variance was used as a criterion for factor selection, assuming a threshold of 10%. For each of the selected factors, we derived a corresponding score considering the items whose factor loading was greater than 0.35. Scores were defined as the weighted average of responses to these items, assuming factor loadings as weights. Histograms were used to describe their empirical distributions. Kernel density estimates and normal densities were superimposed.

Based on the defined scores a non-hierarchical cluster analysis was undertaken. The k-means clustering method was chosen to minimize within-cluster variances. The choice of cluster number was based on the Pseudo F Statistic and Cubic Clustering Criterion.

Statistical analyses were conducted using SAS (version 9.4) and R (version 4.2).

## 3. Results and Discussion

### 3.1. Characteristics of the Survey

Table 1 shows that the socio-demographic characteristics of the respondents are in line with the Italian socio-demographic composition [34] as far as gender, age, and area of residence are concerned, as expected from the sampling procedure. After the weighting of the data, the educational level distribution, in which the higher levels were overrepresented, was also found to be in line with Italian official statistics improving the representativeness of the sample.

### 3.2. Consumers’ Perception of Food Sustainability

The results of the assessment of consumers’ awareness of food sustainability were reported based on the three sections of the questionnaire. Detailed results of all the questions are reported in Table S1 of the Supplementary Materials.

#### 3.2.1. Food Sustainability Knowledge

What emerges from this section is that consumers have little consideration of the impact their food consumption has on the environment and that they perceive sustainable products as too expensive. On the other hand, the reduction of food waste was recognized as a key element for achieving sustainability. In detail, Q1 shows a general lack of awareness regarding the negative effects of respondents’ eating habits on the environment (45%, <4 points on the Likert scale). More than 80% of respondents associate the concept of “sustainable foods” (Q2) with low environmental impact, local food supply chains, and healthy foods. The price of sustainable foods (Q3) is perceived as the biggest barrier to purchase by 79% of respondents, with 61% complaining about the lack of clear nutrition labeling (Table S1).

The lack of awareness regarding the consequences of consumers’ food choices on the environment is supported by several other studies [32,38,39,40,41,42] confirming that consumers underestimate the environmental impacts of animal production [43] and health aspects that are the main determinant for changing consumption habits either in terms of reduction or elimination of meat in the diet [44]. These findings need to be taken into consideration since the sustainability transition requires a changing of the cultural approach of consumers towards a dietary pattern that combines health and sustainable aspects and this could be pursued by public policy united with private-sector proactivity [45].

**Figure 1:**
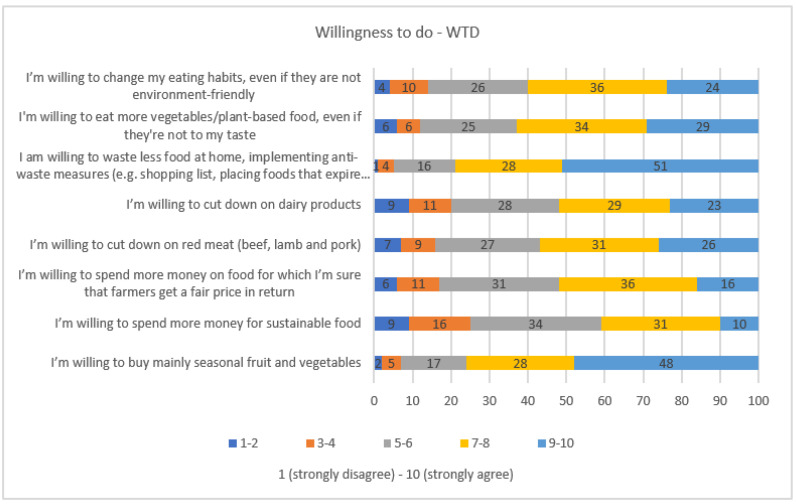
Respondents’ intentions to increase the sustainability of food choices (Willingness to do—WTD) expressed as % values.

In Figure 1, the intention of respondents to actively increase the sustainability level of their dietary pattern is reported. Respondents are found to be willing to act in a more sustainable way (7 to 10 points on the Likert scale) as regards reducing household food waste (79%), consuming more seasonal fruits and vegetables (76%), and eating more plant-based foods (63%). Only 41% of respondents would pay more to buy sustainable food.

#### 3.2.2. Sources of Protein Alternative to Meat

**Figure 2:**
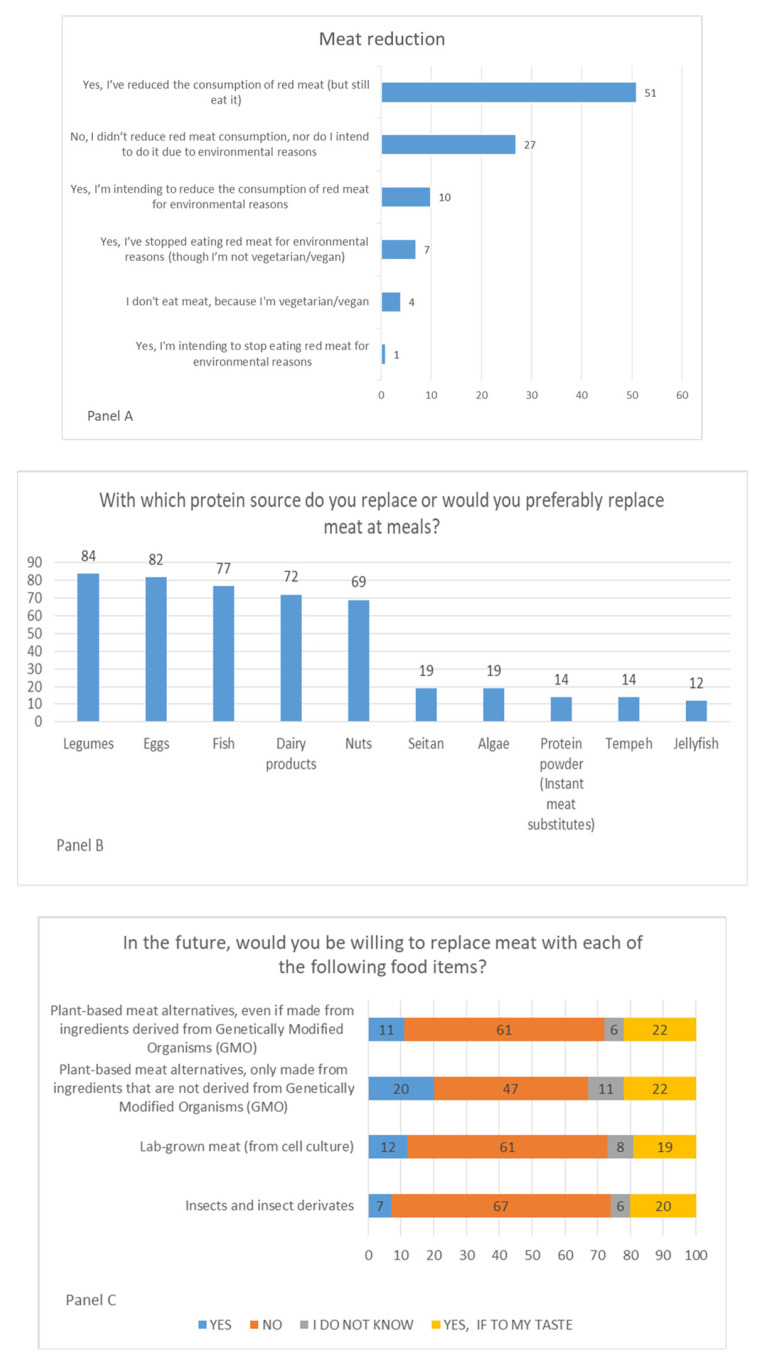
Respondents’ attitudes towards meat and meat alternatives: propensity and reasons for meat reduction (Panel (**A**)); preferred options to replace meat (Panel (**B**)); meat alternatives acceptable for Italians (Panel (**C**)); expressed as % values.

The section of the questionnaire focusing on meat shows that 51% of respondents (Figure 2, panel A) have reduced their consumption for environmental issues but are still eating meat (59% among older people and only 45% in younger people. On the other hand, there is a relevant group of the population (27%) that neither reduced meat consumption nor intended to do so in the future for environmental reasons. Legumes (84%), eggs (82%), and fish (77%) were selected as the preferred protein to replace meat (Figure 2, Panel B). Among novel foods, insects and insect-based products were reported as less accepted alternative sources of proteins (67%); GMO-free plant-based meat alternatives were the most accepted (47%) (Figure 2 Panel C). People with a high level of education, with a high annual income (greater than 36,000 EUR), and students showed a greater propensity to try new foods.

Hence, the alternatives to meat accepted by consumers that could currently be proposed in Italy are the foods typically recommended in the Italian Dietary Guidelines [10], which are legumes, eggs, and fish. Other foods such as insects were strongly rejected by Italian consumers. This could be related to people’s unfamiliarity with novel food (i.e., previous experience, taste expectations, and attitude towards new food experiences) playing an important role in shaping individual inclination [26]. Another interpretation of these findings is the hypothetical nature of the proposed alternative [28]; in other words, cultured meat products and insects are unacceptable to Italian consumers simply because they are not yet available on the market. This aspect is in line with a further result of the present study showing that plant-based meat alternatives (e.g., vegetable burgers) are considered a practicable substitute for meat. These products are well known by consumers and largely accepted, as demonstrated by the rapidly growing sales trend in Italy, other European countries [46], and the United States, where from November 2018 to November 2020, expenditure for plant-based meat alternatives increased from 4.22% to 6.29% [47]. Consumers’ acceptance of plant-based meat alternatives is related to taste, considering an added value the similarity with meat, habit, convenience, and price; on the other hand, negative aspects are found in the sense of “highly processed” products manufactured with “unnatural” methods [23]. In general, consumers consider the production of industrial foods with suspicion, having a strong reliance on what is claimed to be natural—considered de facto better—and this is also related to the lack of awareness and familiarity with technologies used in the production of novel foods [48].

#### 3.2.3. Eating Behaviors: The Importance of Meat and the Request for Rules

**Figure 3:**
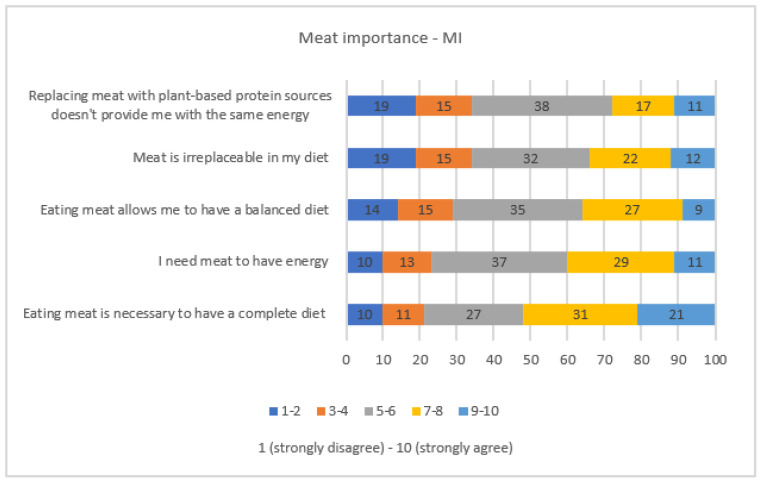
Respondents’ attitudes towards the importance of meat in the diet (Meat Importance—MI) expressed as % values.

Respondents (56%, 7 to 10 points) in particular older age groups (>55 years) consider meat and dairy production less impactful on climate change than deforestation (77%) and exhaust emissions (73%) (Q12—Table S1). As reported in Figure 3, Italians consider meat an important element for a complete (52%, 7 to 10 points on the Likert scale) and balanced diet (36%), and 28% of respondents do not consider vegetable proteins a valid alternative to meat (7–10 points).

**Figure 4:**
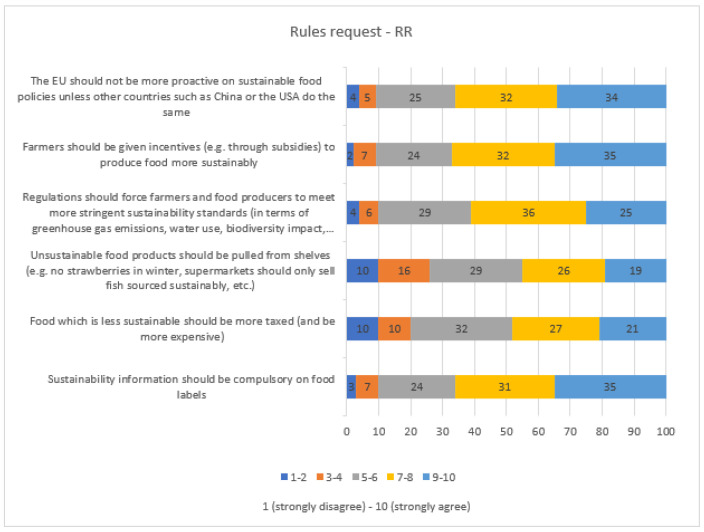
Respondents’ Rules Request—RR for increasing the sustainability of food production expressed as % values.

Meat sounding is not perceived as a problem by respondents and only 19% would prohibit using the word “meat” for plant-origin products (Q10—Table S1). As reported in Figure 4, a large majority of the respondents (61% to 67%) would welcome rules for increasing and promoting the sustainability of food production and food choices. Respondents reported incentives to farmers (67%), sustainability information on labels (66%), a proactive EU sustainable food policy (66%), and stringent sustainability standards for producers (61%) as relevant actions to be put in place with regulations. On the other hand, taxation (48%) and prohibition of selling unsustainable food (45%) were less frequently welcomed as policy actions (Figure 4).

### 3.3. Multivariate Analysis

**Figure 5:**
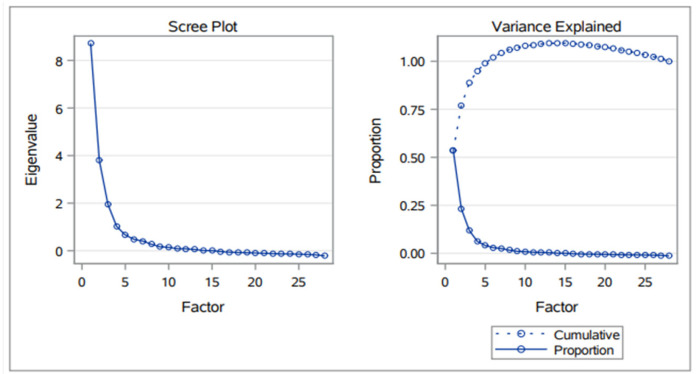
Eigenvalues, proportions, and cumulative proportions of common variance are explained by the 28 factors resulting from the FA.

Factor analysis was undertaken for continuous variables related to responses to questions Q1, Q4, Q8, Q11, and Q12. Figure 5 shows eigenvalues, proportions, and cumulative proportions of common variance explained by the 28 factors resulting from FA. Assuming a threshold of 0.1 for the explained proportion of common variance, the first three factors were selected to account for 88% of the common variance.

After Varimax rotation, for each of the three selected factors, items whose factor loading was greater than 0.35 were identified and reported in Table 2. Based on the selected items, scores were derived as the weighted average of responses to these items, assuming factor loadings as weights.

Factor 1 is the first latent factor that explains more than 53% of the common variability and is identified by the Q8 block of items concerning the relevance of meat. The corresponding score was labeled “Meat Importance” (*MI*) and calculated according to the following formula:

(1) $$MI=\frac{\left(0.84\times Q8.1+0.88\times Q8.2+0.90\times Q8.3+0.86\times Q8.4+0.76\times Q8.5\right)}{\left(0.84+0.88+0.90+0.86+0.76\right)}$$

Factor 2 represents 23% of the common variability and is identified by the Q11 block and by two items of Q12. It expresses respondents’ perceived need for intervention from national and EU institutions to increase and promote the sustainability of food production. The related score was named “Request for Rules” (*RR*) and was calculated according to the following formula:

(2) $$RR=\frac{\left(0.59\times Q11.1+0.70\times Q11.2+0.63\times Q11.3+0.74\times Q11.4+0.66\times Q11.5+0.72\times Q11.6+0.36\times Q12.1+0.50\times Q12.2\right)}{\left(0.59+0.70+0.63+0.74+0.66+0.72+0.36+0.50\right)}$$

Factor 3 shows 12% of the common variability and is defined by the Q4 block. It represents the declared willingness to adopt a more sustainable behavior. It was labeled as “Willingness to do” (*WTD*) and was calculated according to the following formula:

(3) $$WTD=\frac{\left(0.62\times Q4.2+0.59\times Q4.3+0.61\times Q4.4+0.52\times Q4.5+0.38\times Q4.6+0.66\times Q4.7+0.68\times Q4.8+0.38\times Q1.4\right)}{\left(0.62+0.59+0.61+0.52+0.38+0.66+0.68+0.38\right)}$$

**Figure 6:**
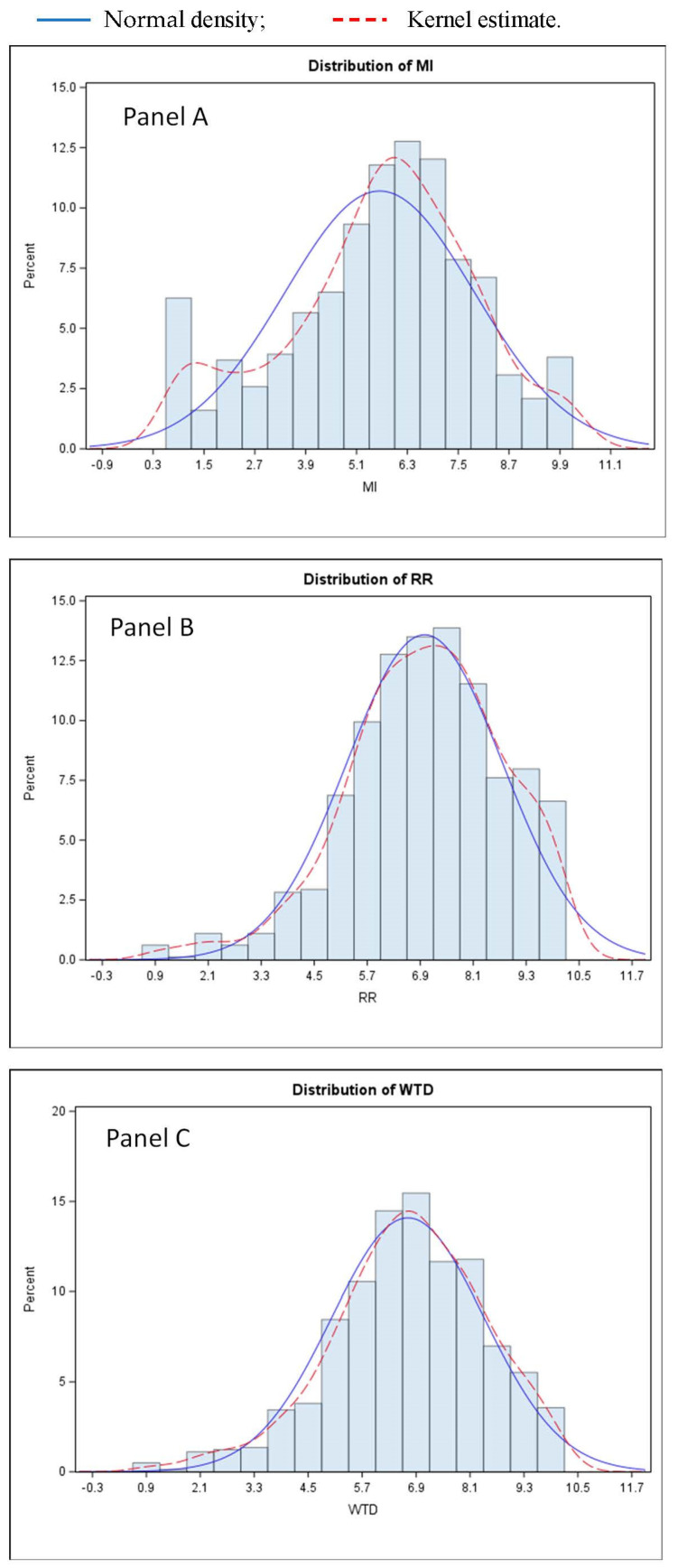
Empirical distributions of the three scores: *MI* (Panel (**A**)), *RR* (Panel (**B**)), and *WTD* (Panel (**C**)).

Empirical distributions of the scores are reported in Figure 6. The *MI* score resulted in the lowest mean value (Mean 5.7; SD 2.2), with a spread distribution of responses and an interesting peak in the areas of very low scores (0.3–1) (Figure 6, panel A). Hence, among the group of Italian consumers who are convinced of the importance of meat, there is a small number for whom meat is not crucial for a healthy and balanced diet. Very relevant in Italy is the request for rules demonstrated by the *RR* empirical distribution that shifts towards high values (Mean 6.9; SD 1.8). A similar shape of the empirical distribution is observed for *WTD* (Mean 6.6; SD 1.7) with an even lower mean value compared to the *RR* distribution.

### 3.4. Cluster Analysis and Socio-Demographic Characteristics of the Resulting Clusters

**Figure 7:**
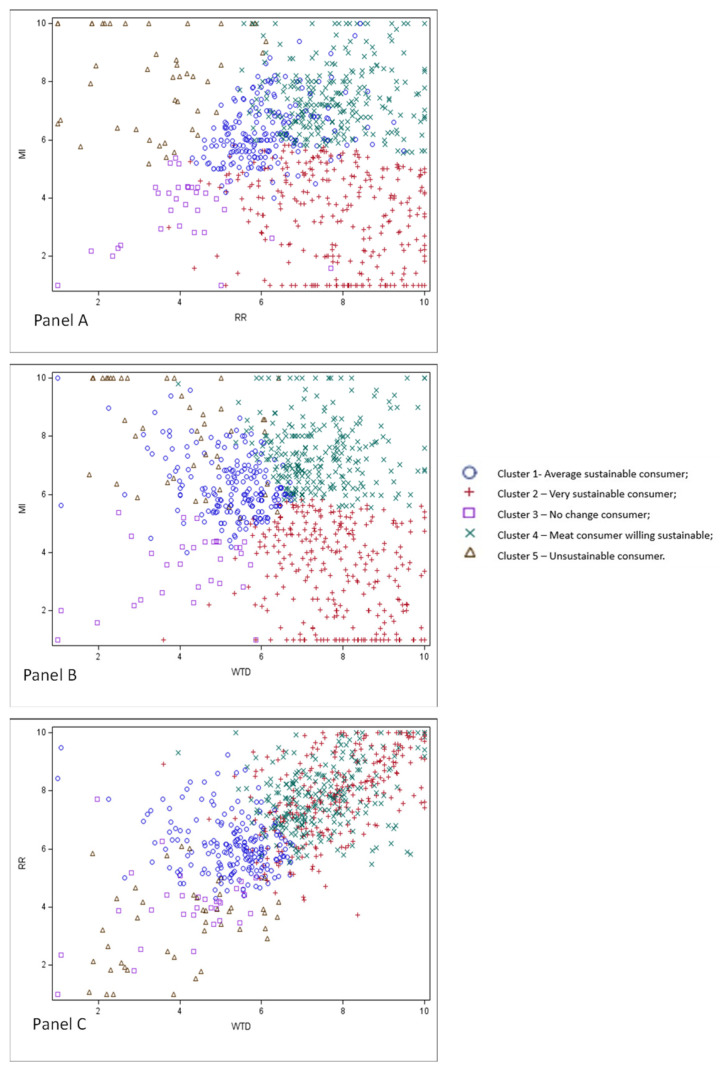
The plots resulting from the cluster analysis: (Panel (**A**))—the intersection between factors *MI* and *RR*; (Panel (**B**))—the cross between factors *MI* and *WTD*; (Panel (**C**))—the cross between factors *RR* and *WTD*.

Based on the proposed scores, a cluster analysis was undertaken. As a result, five clusters with specific characteristics of respondents were identified. Table 3 shows the number of observations per cluster and the clusters’ centroids. The plots resulting from the cluster analysis are reported in Figure 7.

Cluster 1 identifies “Average sustainable consumers” (25.6% of the weighted total); the respondents in this group consider meat important for the diet (*MI* = 6.3), require rules (*RR* = 6.1), and report willingness to do (*WTD* = 5.3) to increase sustainability. However, the attitude of consumers in cluster 1 does not correspond to extreme positions, as seen by the average cluster centroids. In Cluster 1 people over 55 years and with a high level of education appear under-represented (16.1% and 8.1%, respectively) (Table S2).

Cluster 2 identifies “Strongly sustainable consumers” (33% of the weighted total). Meat is not considered very important for this group of people (*MI* = 3.5), but there is a strong demand for rules (*RR* = 7.9) and Cluster 2 respondents have a solid motivation to actively increase the sustainability of food behaviors (*WTD* = 7.8). Cluster 2 is characterized by the presence of older consumers (36.5% of 55–65-year-olds) and families without children (72%) (Table S2).

Cluster 3 includes “No change consumers” (4.4% of the weighted total). These consumers generally have a low score on all factors: they do not consider meat consumption relevant (*MI* = 3.5), do not require rules (*RR* = 4.0), and are unwilling to be proactive for sustainability (*WTD* = 4.1). Men (76.4%), families with children of less than 11 years (61.7%), and homemakers (44.8%) are more distributed in this cluster, living mainly in the northwest or center of Italy (44.4% and 30.5% respectively). Low and high levels of income are under-represented (4.9% and 7.3%) (Table S2).

Cluster 4 identifies “Meat consumers willing to become sustainable” (31.3% of the weighted total). This group considers meat very important for the diet (*MI* = 7.4), strongly welcomes rules (*RR* = 7.7), and wants to change and become more sustainable (*WTD* = 7.4). Compared with total respondents, this cluster is characterized by a relevant percentage of families with children (44.3%) and high levels of income (32.2%) (Table S2).

Cluster 5 corresponds to the “Unsustainable consumers” (5.7% of the weighted total). People in this group consider meat essential for the diet (*MI* = 8.0), do not welcome rules (*RR* = 3.5), and are unwilling to act for sustainability (*WTD* = 4.1). Respondents are mainly men (66%), living in the northern regions of Italy (61.3%), 17% have a low educational level and 37.9% have an annual income above 36,000 Euros (Table S2).

The cluster analysis confirmed the importance of meat for Italian consumers emphasizing other aspects of the consumer’s approach to meat and the sustainability of food choices. Almost one-fourth of respondents, identified by Cluster 1—Average sustainable consumer, consider meat very important. However, these consumers are also open to change and welcome rules from policymakers to increase sustainable food choices. In addition, two out of five Clusters (i.e., Clusters 2 and 3, covering about 38% of the total) considered meat replaceable in the diet, and more than half of respondents stated they had reduced but not eliminated meat for environmental reasons. As pointed out in several recent studies [49,50,51], meat has acquired a negative image mainly because of its association with environmental issues [50], as well as religious, ethical, and moral concerns [49,52]. Whereas the negative attitude towards meat is not necessarily associated with a reduction of meat consumption [53]. The majority of respondents (61% of respondents, corresponding to clusters 2 and 4) have a strong motivation for sustainability (high *WTD* and *RR*); however, the two clusters are differentiated by their consideration of meat: not very relevant for Cluster 2 (Strongly sustainable consumer—corresponding to 33% of respondents), very important for Cluster 4 (meat consumer willing to be sustainable—corresponding to 31.3% of respondents). The ‘No-change consumers’ group (i.e., Cluster 3) and the ‘Unsustainable consumers’ (i.e., Cluster 5) cover the 10% of the population characterized by a general lack of interest in the issue of sustainability. One of the research questions of this work was related to the identification of consumers’ policy supports that could be helpful in increasing the sustainability of food choices. This study shows that Italian consumers would welcome rules for more information on products (e.g., clear labels). Taxation and other forms of prohibition have been flagged as less acceptable, probably due to fears of rising prices that have been recognized as a barrier to the selection of sustainable foods (perceived as expensive). At the same time, the large majority (90%) of respondents requests rules by policymakers and recognize individual responsibility (*WTD*) to improve the sustainability of dietary choices (Clusters 1, 2, and 4). Similar results emerged from Whittall et al. [54] when reporting that consumers are willing to adopt sustainability despite uncertainty about what action should be taken.

The socio-demographic characteristics of respondents influence the sustainability of food choices, confirming current literature on the topic [55,56,57]. In particular, according to Verain and Dagevos [57], men have a greater propensity to consume meat than women, in line with the present study in which men are most commonly found in the clusters that consider meat important (76.4% of respondents in Cluster 3 and 66% of respondents in Cluster 5 vs. 50% of the total population). Besides the specific aspect of meat preference and importance, several studies [55,56,57] reported women as more interested in sustainability than men. In terms of age, in the present study, the oldest group (55–65 years) showed more attention to sustainability and did not perceive meat as an important element of the diet.

The presence of children in the household is a determining factor for meat consumption and represents a barrier to the replacement of meat with other foods. Families with young children (under 11 years) were most reluctant to be sustainable, being more frequent in Cluster 3 “Consumer against change” and in Cluster 4 characterized by consumers that consider meat important. To complete the picture, it should be pointed out that the two clusters corresponding to consumers with a general lack of interest in sustainability (Cluster 3—the no-change consumers’ group and Cluster 5—the unsustainable consumers) mainly include men, from northern regions, aged over 55 years, with low educational levels and a medium income. These findings are confirmed by Neuhofer & Lusk [47] reporting that the buyers of plant-based meat alternatives that could be considered an element of a sustainable attitude, tend to be young, single, female, highly educated, employed, and with a higher income. The socio-demographic profiling that emerged in the cluster characterization of the present work has several similarities with the results reported by Gutiérrez-Villar et al. [58] in Spanish households in which sustainable dietary habits are frequently found in high-income groups, families without children, and people living in small cities.

## 4. Conclusions

The main result of this paper is related to the role of meat in the diet as the key aspect characterizing the respondents and marking the differences between population groups. Almost half (45%) of respondents have a general lack of awareness regarding the consequences of their food choices on the environment and consider meat an important element of the diet often claimed as not replaceable. Meat consumption represents a challenge to human health and the environment, and its reduction is a positive and achievable goal [59,60,61,62,63,64,65,66]. Recently, IPCC [63] has reinforced the concept previously expressed by the EAT-Lancet Commission [61] underlining how a plant-based diet represents the best choice for people and the environment. Dietary patterns are directly related to overall food demand, thus diets can be an important entry point for action and best practices to be put in place both individually and collectively to improve the sustainability of food systems [64,65]. However, the substitution of meat is a complex behavioral change as it is related to the sensory experience of eating meat, the taste, and subjective concerns about the risk of protein deficiency [66]. The most relevant novelty of this assessment is that besides clarifying Italian consumers’ view of the importance of meat, there is room for recommendations for reducing the consumption of this food item. However, it should be considered that the motivation for the reduction of meat consumption is more related to health factors than environmental reasons. A key point of consumers’ attitude towards meat is related to the possible alternatives, meaning legumes, eggs, dairy products, and fish that should be considered in terms of practical applicable recommendations. In view of the importance of increasing the vegetable sources of proteins for human health and the environment [67], the use of plant-based meat alternatives could be a way to increase the choices of legumes in forms acceptable to consumers thus increasing the variety of food choices. The future development of dietary guidelines should consider these environmental and societal impacts, as well as issues related to health [68]. However, further studies are necessary to provide a more detailed understanding of people’s preferences towards new sustainable foods, for example, proposing experimentation with novel foods (e.g., insects) in terms of sensory experience. However, changing consumer preferences and introducing new foods requires time without any assurance of success. An important finding of this study is related to the fact that consumers declared a high interest in rules from policymakers and this aspect needs to be considered in the framework of policy strategies and approaches.

This study has strengths and limitations. The most important strength of the work is the use, for the assessment, of a proven questionnaire adapted to the local (Italian) context [37]. This is particularly relevant given the fact that the sustainability of diet is an emerging topic only recently addressed in the framework of nutritional recommendations and dietary guidelines [10]. The added value of this approach is that the assessment of consumers’ perceptions of the various aspects of food sustainability was preceded by a validation process reinforcing the value of the present assessment. Another strength of the present work is linked to the sampling procedure that allowed for a group of respondents as far as possible representative of the Italian population as regards area of residence, age groups, gender, and educational level (after the weighting procedure). This is an important point since the results of this work could be capitalized on through recommendations that combine nutritional and sustainability considerations increasing the applicability of the findings. However, the sampling procedure also represents a limitation since the representativeness for a well-defined set of variables is not guaranteed for other variables not included in the selection process such as income, family size, household composition, etc. In this sense, the decision to include people with the ability to use online tools excluded the elderly, who are therefore not represented in the assessment. Perhaps the most important limitation of the work is the fact that the evaluation is based on self-declared behaviors and intentions of change that might not fully reflect reality [69].

## Figures and Tables

**Table 1:** Population socio-demographic information and body mass index (BMI) with weight.

Gender		
Man	407.05	49.94%
Woman	407.95	50.06%
Age		
Mean = 43.43 SD = 12.68		
Age groups		
18–35	197.42	26.07%
35–45	170.09	28.87%
45–55	213.80	26.23%
55–65	218.65	26.83%
Family size		
1	85.72	10.52%
2	222.20	27.26%
3	232.41	28.52%
4	210.31	25.80%
5	50.36	6.18%
6	5.66	0.7%
>6	8.33	1.02%
Presence of children in the family		
Age ≤ 11 years	178.91	21.95%
Age > 11 years	130.45	16.01%
No	505.63	62.04%
Education level		
Low (primary school or lower)	62.73	7.70%
Middle-low (secondary school)	525.04	64.42%
High-middle (first university level)	107.42	13.18%
High (university degree or higher)	119.80	14.7%
Area of origin		
Northwest Italy	216.89	26.61%
Northeast Italy	158.86	19.49%
Central Italy	161.71	19.84%
South Italy	188.69	23.16%
Island	88.84	10.9%
Household income		
<18,000 EUR	157.23	21.56%
[18,000 EUR, 36,000 EUR]	366.33	50.25%
>36,000 EUR	205.51	28.19%
Body mass index		
Underweight	32.10	3.94%
Normal weight	465.89	57.16%
Overweight	221.57	27.19%
Obese	95.43	11.71%
Working activity		
Student	66.40	8.15%
Housemaker	85.62	10.51%
Retired	33.61	4.12%
Unemployed or looking for a first job	57.84	7.09%
Manual worker	101.35	12.44%
Employee	295.60	36.27%
Self-employed	130.03	15.96%
Others	44.54	5.46%
Urban		
<100,000	638.03	78.29%
≥100,000	176.96	21.71%

**Table 2:** Factor loading after Varimax rotation, corresponding factor, and commonality.

Items Group	Questions	Factor Loading	Communality	Factor
Meat Importance (*MI*)				
Q8.1	Eating meat is necessary to have a complete diet	0.84	0.763	1
Q8.2	I need meat to have energy	0.88	0.781	1
Q8.3	Eating meat allows me to have a balanced diet	0.9	0.812	1
Q8.4	Meat is irreplaceable in my diet	0.86	0.776	1
Q8.5	Replacing meat with plant-based protein sources doesn’t provide me with the same energy	0.76	0.594	1
Request for Rules (*RR*)				
Q11.1	Sustainability information should be compulsory on food labels	0.59	0.67	2
Q11.2	Food that is less sustainable should be taxed higher (and be more expensive)	0.7	0.637	2
Q11.3	Unsustainable food products should be removed from shelves (e.g., no strawberries in winter, supermarkets should sell only sourced sustainably fish, etc.)	0.63	0.48	2
Q11.4	Regulations should force farmers and food producers to meet more stringent sustainability standards (in terms of greenhouse gas emissions, water use, biodiversity, etc.)	0.74	0.7	2
Q11.5	Farmers should be given incentives (e.g., through subsidies) to produce food more sustainably	0.66	0.686	2
Q11.6	The EU should not be more proactive on sustainable food policies unless other countries such as China or the USA do the same	0.72	0.702	2
Q12.1	Emissions from aircraft, trains, cars, trucks and ships	0.36	0.626	2
Q12.2	The production of meat and dairy products, which we eat and drink	0.5	0.471	2
Willingness to do (*WTD*)				
Q4.2	I’m willing to spend more money on sustainable food	0.62	0.586	3
Q4.3	I’m willing to spend more money on food for which I’m sure farmers get a fair price for in return	0.59	0.512	3
Q4.4	I’m willing to cut down on red meat (beef, lamb and pork)	0.61	0.615	3
Q4.5	I’m willing to cut down on dairy	0.52	0.387	3
Q4.6	I am willing to waste less food at home, implementing anti-waste measures (e.g., shopping lists, placing foods that expire first at the front of the refrigerator, etc.	0.38	0.545	3
Q4.7	I’m willing to eat more vegetables/plant-based food, even if they’re not to my taste	0.66	0.598	3
Q4.8	I’m willing to change my eating habits, even if they are not environment-friendly	0.68	0.725	3
Q1.4	Sustainability issues influence my food choices	0.38	0.775	3

**Table 3:** Cluster results: centroids and number of observations.

Cluster	Weighted Obs (%)	Cluster Centroids Means (SD)		
		*MI*	*RR*	*WTD*
1—Average sustainable consumer	208.9 (25.6%)	6.28 (1.04)	6.07 (0.98)	5.28 (0.96)
2—Strongly sustainable consumer	268.9 (33%)	3.45 (1.53)	7.92 (1.39)	7.79 (1.19)
3—No change in consumers	35.5 (4.4%)	3.51 (1.24)	3.96 (1.32)	4.13 (1.49)
4—Meat consumers willing to be sustainable	254.8 (31.3%)	7.40 (1.14)	7.68 (1.08)	7.37 (0.97)
5—Unsustainable consumers	46.9 (5.7%)	8.02 (1.64)	3.53 (1.38)	4.09 (1.46)

## Supplementary Materials

The following supporting information can be downloaded at: https://www.mdpi.com/article/10.3390/nu15183861/s1, Table S1: Answers to all the questions (%); Table S2: Socio-demographic characteristics of the 5 clusters (number of weighted observations).

## Abbreviations

GHGE	Greenhouse Gas Emissions
WHO	World Health Organization
FAO	Food and Agriculture Organization
GMO	Genetically Modified Organisms
MD	Mediterranean Diet
CAWI	Computer Assisted Web Interviewing
ISTAT	National Institute of Statistics
SD	Standard Deviation
FA	Factor Analysis
PCA	Principal Component Analysis
CI	Confidence Interval
MI	Meat Importance
RR	Request for Rules
WTD	Willing to do

## References

[1] United Nations (2022). World Population Prospects 2022: Summary of Results.

[2] Poore J., Nemecek T. (2018). Reducing Food’s Environmental Impacts through Producers and Consumers. Science.

[3] Ritchie H., Roser M. Environmental Impacts of Food Production. https://ourworldindata.org/environmental-impacts-of-food#citation.

[4] Gerber P.J., FAO (2013). Tackling Climate Change through Livestock: A Global Assessment of Emissions and Mitigation Opportunities.

[5] Nelson M.E., Hamm M.W., Hu F.B., Abrams S.A., Griffin T.S. (2016). Alignment of Healthy Dietary Patterns and Environmental Sustainability: A Systematic Review. Adv. Nutr..

[6] Pratesi I., Alessi E. (2021). Dalle Pandemie Alla Perdita di Biodiversità: Dove ci sta Portando il Consumo di Carne.

[7] González-García S., Esteve-Llorens X., Moreira M.T., Feijoo G. (2018). Carbon Footprint and Nutritional Quality of Different Human Dietary Choices. Sci. Total Environ..

[8] FAO WHO Sustainable Healthy Diets: Guiding Principles. http://www.fao.org/3/ca6640en/ca6640en.pdf.

[9] UN The Sustainable Development Agenda. https://sdgs.un.org/goals.

[10] Rossi L., Berni Canani S., Censi L., Gennaro L., Leclercq C., Scognamiglio U., Sette S., Ghiselli A. (2022). The 2018 Revision of Italian Dietary Guidelines: Development Process, Novelties, Main Recommendations, and Policy Implications. Front. Nutr..

[11] Wang F., Zheng J., Yang B., Jiang J., Fu Y., Li D. (2015). Effects of Vegetarian Diets on Blood Lipids: A Systematic Review and Meta-Analysis of Randomized Controlled Trials. JAHA.

[12] Sutliffe J.T., Fuhrman J.H., Carnot M.J., Beetham R.M., Peddy M.S. (2016). Nutrient-Dense, Plant-Rich Dietary Intervention Effective at Reducing Cardiovascular Disease Risk Factors for Worksites: A Pilot Study. Altern. Ther. Health Med..

[13] Satija A., Hu F.B. (2018). Plant-Based Diets and Cardiovascular Health. Trends Cardiovasc. Med..

[14] Kahleova H., Levin S., Barnard N. (2017). Cardio-Metabolic Benefits of Plant-Based Diets. Nutrients.

[15] Naghshi S., Sadeghi O., Willett W.C., Esmaillzadeh A. (2020). Dietary Intake of Total, Animal, and Plant Proteins and Risk of All Cause, Cardiovascular, and Cancer Mortality: Systematic Review and Dose-Response Meta-Analysis of Prospective Cohort Studies. BMJ.

[16] Huang J., Liao L., Weinstein S., Sinha R., Graubard B., Albanes D. (2020). Association Between Plant and Animal Protein Intake and Overall and Cause-Specific Mortality. JAMA Intern. Med..

[17] Xiang-Xiu Q. (2020). Associations of Dietary Protein Intake with All-Cause, Cardiovascular Disease, and Cancer Mortality: A Systematic Review and Meta-Analysis of Cohort Studies. Nutr. Metab. Cardiovasc. Dis..

[18] Springmann M., Godfray H.C.J., Rayner M., Scarborough P. (2016). Analysis and Valuation of the Health and Climate Change Cobenefits of Dietary Change. Proc. Natl. Acad. Sci. USA.

[19] Stagnari F., Maggio A., Galieni A., Pisante M. (2017). Multiple Benefits of Legumes for Agriculture Sustainability: An Overview. Chem. Biol. Technol. Agric..

[20] Guiguitant J., Vile D., Ghanem M.E., Wery J., Marrou H. (2020). Evaluation of Pulse Crops’ Functional Diversity Supporting Food Production. Sci. Rep..

[21] Rumpold B.A., Schlüter O. (2015). Insect-Based Protein Sources and Their Potential for Human Consumption: Nutritional Composition and Processing. Anim. Front..

[22] van Huis A., Oonincx D.G.A.B. (2017). The Environmental Sustainability of Insects as Food and Feed. A Review. Agron. Sustain. Dev..

[23] Rubio N.R., Xiang N., Kaplan D.L. (2020). Plant-Based and Cell-Based Approaches to Meat Production. Nat. Commun..

[24] Bhat Z.F., Kumar S., Bhat H.F. (2017). In Vitro Meat: A Future Animal-Free Harvest. Crit. Rev. Food Sci. Nutr..

[25] Basile A., Ferranti P. (2019). Synthetic Meat: Acceptance. Encycl. Food Secur. Sustain..

[26] Palmieri N., Perito M., Lupi C. (2020). Consumer Acceptance of Cultured Meat: Some Hints from Italy. Br. Food J..

[27] WHO Food Safety Aspects of Cell-Based Food. http://www.fao.org/documents/card/en/c/cc4855en.

[28] Post M.J., Levenberg S., Kaplan D.L., Genovese N., Fu J., Bryant C.J., Negowetti N., Verzijden K., Moutsatsou P. (2020). Scientific, Sustainability and Regulatory Challenges of Cultured Meat. Nat. Food.

[29] McClements D.J., Grossmann L. (2021). A Brief Review of the Science behind the Design of Healthy and Sustainable Plant-Based Foods. NPJ Sci. Food.

[30] Kumar P., Chatli M.K., Mehta N., Singh P., Malav O.P., Verma A.K. (2017). Meat Analogues: Health Promising Sustainable Meat Substitutes. Crit. Rev. Food Sci. Nutr..

[31] Roselli L., Stasi A., Seccia A. (2006). Atteggiamento dei consumatori nei confronti dell’evoluzione del sistema agro-alimentare: L’introduzione di alimenti geneticamente modificati. Econ. Agro-Aliment..

[32] Hartmann C., Siegrist M. (2017). Consumer Perception and Behaviour Regarding Sustainable Protein Consumption: A Systematic Review. Trends Food Sci. Technol..

[33] Coderoni S., Perito M.A., Cardillo C. (2017). Consumer Behaviour in Italy. Who Spends More to Buy a Mediterranean Diet?. New Medit.

[34] ISTAT ISTAT—Popolazione 15 Anni e Oltre per Titolo di Studio—Regolamento Precedente (Fino al 2020). http://dati.istat.it/Index.aspx?DataSetCode=DCCV_POPTIT1_UNT2020.

[35] World Medical Association Wma Declaration of Helsinki—Ethical Principles for Medical Research Involving Human Subjects. https://www.wma.net/policies-post/wma-declaration-of-helsinki-ethical-principles-for-medical-research-involving-human-subjects/.

[36] SWG (2021). Modello Di Organizzazione Gestione e Controllo 2021.

[37] Aureli V., Nardi A., Peluso D., Scognamiglio U., Rossi L. (2022). Consumers’ Attitude towards Sustainability in Italy: Process of Validation of a Duly Designed Questionnaire. Foods.

[38] Dornhoff M., Hörnschemeyer A., Fiebelkorn F. (2020). Students’ Conceptions of Sustainable Nutrition. Sustainability.

[39] Hoek A.C., Pearson D., James S.W., Lawrence M.A., Friel S. (2017). Shrinking the Food-Print: A Qualitative Study into Consumer Perceptions, Experiences and Attitudes towards Healthy and Environmentally Friendly Food Behaviours. Appetite.

[40] Lea E., Worsley A. (2008). Australian Consumers’ Food-Related Environmental Beliefs and Behaviours. Appetite.

[41] Macdiarmid J.I., Douglas F., Campbell J. (2016). Eating like There’s No Tomorrow: Public Awareness of the Environmental Impact of Food and Reluctance to Eat Less Meat as Part of a Sustainable Diet. Appetite.

[42] Mann D., Thornton L., Crawford D., Ball K. (2018). Australian Consumers’ Views towards an Environmentally Sustainable Eating Pattern. Public Health Nutr..

[43] Vanhonacker F., Van Loo E.J., Gellynck X., Verbeke W. (2013). Flemish Consumer Attitudes towards More Sustainable Food Choices. Appetite.

[44] Latvala T., Niva M., Mäkelä J., Pouta E., Heikkilä J., Kotro J., Forsman-Hugg S. (2012). Diversifying Meat Consumption Patterns: Consumers’ Self-Reported Past Behaviour and Intentions for Change. Meat Sci..

[45] Moberg E., Allison E., Harl H., Arbow T., Almaraz M., Dixon J., Scarborough C., Skinner T., Rasmussen L., Salter A. (2021). Combined Innovations in Public Policy, the Private Sector and Culture Can Drive Sustainability Transitions in Food Systems. Nat. Food.

[46] GFI Europe Plant-Based Food Retailer Market Insights 2020–2022. https://gfieurope.org/wp-content/uploads/2023/03/2020-2022-Europe-retail-market-insights.pdf.

[47] Neuhofer Z.T., Lusk J.L. (2022). Most Plant-Based Meat Alternative Buyers Also Buy Meat: An Analysis of Household Demographics, Habit Formation, and Buying Behavior among Meat Alternative Buyers. Sci. Rep..

[48] Siegrist M., Hartmann C. (2020). Consumer Acceptance of Novel Food Technologies. Nat. Food.

[49] Berndsen M., van der Pligt J. (2005). Risks of Meat: The Relative Impact of Cognitive, Affective and Moral Concerns. Appetite.

[50] Povey R., Wellens B., Conner M. (2001). Attitudes towards Following Meat, Vegetarian and Vegan Diets: An Examination of the Role of Ambivalence. Appetite.

[51] Font-i-Furnols M., Guerrero L. (2014). Consumer Preference, Behavior and Perception about Meat and Meat Products: An Overview. Meat Sci..

[52] Troy D.J., Kerry J.P. (2010). Consumer Perception and the Role of Science in the Meat Industry. Meat Sci..

[53] Holm L., Møhl M. (2000). The Role of Meat in Everyday Food Culture: An Analysis of an Interview Study in Copenhagen. Appetite.

[54] Whittall B., Warwick S.M., Guy D.J., Appleton K.M. (2023). Public Understanding of Sustainable Diets and Changes towards Sustainability: A Qualitative Study in a UK Population Sample. Appetite.

[55] Clonan A., Wilson P., Swift J.A., Leibovici D.G., Holdsworth M. (2015). Red and Processed Meat Consumption and Purchasing Behaviours and Attitudes: Impacts for Human Health, Animal Welfare and Environmental Sustainability. Public Health Nutr..

[56] Tobler C., Visschers V.H.M., Siegrist M. (2011). Eating Green. Consumers’ Willingness to Adopt Ecological Food Consumption Behaviors. Appetite.

[57] Verain M.C.D., Dagevos H. (2022). Comparing Meat Abstainers with Avid Meat Eaters and Committed Meat Reducers. Front. Nutr..

[58] Gutiérrez-Villar B., Melero-Bolaños R., Montero-Simo M.J., Araque-Padilla R.A. (2022). Profiling Consumers with an Environmentally Sustainable and Healthy Diet: The Case of Spanish Households. Front. Nutr..

[59] Perignon M., Vieux F., Soler L.-G., Masset G., Darmon N. (2017). Improving Diet Sustainability through Evolution of Food Choices: Review of Epidemiological Studies on the Environmental Impact of Diets. Nutr. Rev..

[60] Steenson S., Buttriss J.L. (2021). Healthier and More Sustainable Diets: What Changes Are Needed in High-Income Countries?. Nutr. Bull..

[61] Willett W., Rockström J., Loken B., Springmann M., Lang T., Vermeulen S., Garnett T., Tilman D., DeClerck F., Wood A. (2019). Food in the Anthropocene: The EAT—Lancet Commission on Healthy Diets from Sustainable Food Systems. Lancet.

[62] Tiberius V., Borning J., Seeler S. (2019). Setting the Table for Meat Consumers: An International Delphi Study on in Vitro Meat. NPJ Sci. Food.

[63] IPCC Climate Change and Land—Summary for Policymakers. https://www.ipcc.ch/site/assets/uploads/sites/4/2019/12/02_Summary-for-Policymakers_SPM.pdf.

[64] Meybeck A., Gitz V. (2017). Sustainable Diets within Sustainable Food Systems. Proc. Nutr. Soc..

[65] Sun Z., Scherer L., Tukker A., Spawn-Lee S.A., Bruckner M., Gibbs H.K., Behrens P. (2022). Dietary Change in High-Income Nations Alone Can Lead to Substantial Double Climate Dividend. Nat. Food.

[66] Humpenöder F., Bodirsky B.L., Weindl I., Lotze-Campen H., Linder T., Popp A. (2022). Projected Environmental Benefits of Replacing Beef with Microbial Protein. Nature.

[67] Ferrari L., Panaite S.-A., Bertazzo A., Visioli F. (2022). Animal- and Plant-Based Protein Sources: A Scoping Review of Human Health Outcomes and Environmental Impact. Nutrients.

[68] Tong T.Y.N., Papier K., Key T.J. (2022). Meat, Vegetables and Healt—Interpreting the Evidence. Nat. Med..

[69] Wright K.B. (2005). Researching Internet-Based Populations: Advantages and Disadvantages of Online Survey Research, Online Questionnaire Authoring Software Packages, and Web Survey Services. J. Comput.-Mediat. Commun..

