# Supplementary material for: Sustainability Perception of Italian Consumers: Is it Possible to Replace Meat, and What Is the Best Alternative?
Source: Nutrients. 2023 Sep 5;15(18):3861. doi: 10.3390/nu15183861 (PMC10650016; doi:10.3390/nu15183861)
Supplement: Supplementary file 1 [file nutrients-15-03861-s001.zip › nutrients-2594344-supplementary.pdf]

Supplementary Materials: Table S1 - Answers to all the questions (%)

**Q1. To what extent do you agree with each of the following statements? [answer from 1 (strongly disagree) to 10 (strongly agree)] (%)**	**1-2**	**3-4**	**5-6**	**7-8**	**9-10**
Q1.1 My food habits negatively affect the environment	23	22	34	16	5
Q1.2 When compared to car use, food habits have only little impact on the environment	12	19	38	22	9
Q1.3 I pay attention to the impact that my food choices have on the environment.	7	13	36	30	14
Q1.4 Sustainability issues influence my food choices.	10	15	34	30	11
Q1.5 I improved my habits in favor of food sustainability, following the government's advertising campaigns.	16	16	33	26	9
Q1.6 I do not want someone to tell me or decide for me what I should eat or not	10	13	35	24	18

**Q2. What comes to your mind when thinking about "sustainable" food? (%)**	**Yes**
Q2.1 Low environmental impact	89
Q2.2 Availability and affordability of food for all	58
Q2.3 Use of pesticides and GMOs to be avoided	79
Q2.4 Local supply chains	81
Q2.5 Fair revenue for farmers	69
Q2.6 High animal welfare standards	79
Q2.7 Economic growth in the agri-food sector	63
Q2.8 Minimally processed, traditional	78
Q2.9 Healthy	81

**Q3. What are the main reasons preventing you from eating (more) sustainably? (%)**	**Yes**
Lack of information on how to do so	54
Lack of clear labelling	61
I'm not concerned with sustainability	41
Lack of sustainable food products in my usual shopping places / eating places	55
Too expensive	79
I'm not willing to change my eating habits	40
Lack of time (to buy it, to cook it, etc.)	49

**Q4. To what extent do you agree with each of the following statements? [answer from 1 (strongly disagree) to 10 (strongly agree)] (%)**	**1-2**	**3-4**	**5-6**	**7-8**	**9-10**
Q4.1 I'm willing to buy mainly seasonal fruit and vegetables	2	5	17	28	48
Q4.2 I'm willing to spend more money for sustainable food	9	16	34	31	10
Q4.3 I'm willing to spend more money on food for which I'm sure that farmers get a fair price in return	6	11	31	36	16
Q4.4 I'm willing to cut down on red meat (beef, lamb and pork)	7	9	27	31	26
Q4.5 I'm willing to cut down on dairy	9	11	28	29	23
Q4.6 I am willing to waste less food at home, implementing anti-waste measures (e.g. shopping list, placing foods that expire first in front of the refrigerator, etc.	1	4	16	28	51
Q4.7 I'm willing to eat more vegetables/plant-based food, even if they're not to my taste	6	6	28	34	29
Q4.8 I'm willing to change my eating habits, even if they are not environment-friendly	4	10	26	36	24

**Q5. Have you reduced (or plans to reduce) the consumption of red meat (beef, lamb and pork) for environmental reasons? (Tick only one answer) (%)**	**Yes**

Q5.1 I don't eat meat, because I'm vegetarian/vegan	4				
Q5.2 Yes, I've stopped eating red meat for environmental reasons (though I'm not vegetarian/vegan)	7				
Q5.3 Yes, I've reduced the consumption of red meat (but still eat it)	51				
Q5.4 Yes, I'm intending to reduce the consumption of red meat for environmental reasons	10				
Q5.5 Yes, I'm intending to stop eating red meat for environmental reasons	1				
Q5.6 No, I didn't reduce red meat consumption, nor do I intend to do it due to environmental reasons	27				
**Q6. With which protein source do you replace or would you preferably replace meat at meals? (%)**	**Yes**				
Q6.1 Fish	77				
Q6.2 Eggs	82				
Q6.3 Dairy products	72				
Q6.4 Protein powder (Instant meat substitutes)	14				
Q6.5 Legumes	84				
Q6.6 Nuts	69				
Q6.7 Seitan	19				
Q6.8 Tempeh	14				
Q6.9 Algae	19				
Q6.10 Jellyfish	12				
**Q7. In the future, would you be willing to replace meat with each of the following food items? (%)**	**Yes**	**Yes, if to my taste**	**I do not know**	**No**	
Q7.1 Insects and insect derivates	7	6	67	20	
Q7.2 Lab-grown meat (from cell culture)	12	8	61	19	
Q7.3 Plant-based meat alternatives, only made from ingredients that are not derived from Genetically Modified Organisms (GMO)	20	11	47	22	
Q7.4 Plant-based meat alternatives, even if made from ingredients derived from Genetically Modified Organisms (GMO)	11	6	61	22	
**Q8. To what extent do you agree with each of the following statements? [answer from 1 (strongly disagree) to 10 (strongly agree)] (%)**	**1-2**	**3-4**	**5-6**	**7-8**	**9-10**
Q8.1 Eating meat is necessary to have a complete diet	10	11	27	31	21
Q8.2 I need meat to have energy	14	15	35	27	9
Q8.3 Eating meat allows me to have a balanced diet	10	13	37	29	11
Q8.4 Meat is irreplaceable in my diet	19	15	32	22	12
Q8.5 Replacing meat with plant-based protein sources doesn't provide me with the same energy	19	15	38	17	11
**Q9. If all meat products comply with farm animal welfare rules and prices were to increase by 30% compared to current prices (Tick only one answer) (%)**	**Yes**				
Q9.1 I would eat the same amount of meat products as I currently do	42				
Q9.2 I would eat more meat products than I currently do	13				
Q9.3 I would eat less meat products than I currently do	45				
**Q10. To what extent do you agree that companies use meat-related names like 'sausage' and 'burger' to describe meat-free vegetarian products (e.g. a veggie 'burger')? (Tick only one answer) (%)**	**Yes**				
Q10.1 It should never be allowed for vegetarian products	19				
Q10.2 It should be allowed only if it is clearly labelled it's a vegetarian product	44				
Q10.3 I don't see any problem for using such names	23				
Q10.4 I have no opinion	14				
**Q11. To what extent do you agree with each of the following statements? [answer from 1 (strongly disagree) to 10 (strongly agree)] (%)**	**1-2**	**3-4**	**5-6**	**7-8**	**9-10**

Q11.1 Sustainability information should be compulsory on food labels	3	7	24	31	35
Q11.2 Food which is less sustainable should be more taxed (and be more expensive)	10	10	32	27	21
Q11.3 Unsustainable food products should be pulled from shelves (e.g. no strawberries in winter, supermarkets should only sell fish sourced sustainably, etc.)	10	16	29	26	19
Q11.4 Farmers should be given incentives (e.g. through subsidies) to produce food more sustainably	2	7	24	32	35
Q11.5 The EU should not be more proactive on sustainable food policies unless other countries such as China or the USA do the same	4	5	25	32	34
Q11.6 Regulations should force farmers and food producers to meet more stringent sustainability standards (in terms of greenhouse gas emissions, water use, biodiversity impact, etc.)	4	6	29	36	25
**Q12. 12. To what extent do the following activities contribute to climate change? [answer from 1 (strongly disagree) to 10 (strongly agree)] (%)**	**1-2**	**3-4**	**5-6**	**7-8**	**9-10**
Q12.1 Emissions from aircraft, trains, cars, trucks and ships	3	7	17	30	43
Q12.2 The production of meat and dairy products, which we eat and drink	5	9	30	34	22
Q12.3 The felling of trees and forests	2	4	17	28	49

Supplementary Materials: Table S2 – Socio-demographic characteristics of the 5 clusters (number of weighted observations)

	Categories	Cluster 1	Cluster 2	Cluster 3	Cluster 4	Cluster 5	Total
**Gender**	Man	47.55	45.66	76.40	49.80	65.97	49.94
	Woman	52.45	54.34	23.60	50.20	34.03	50.06
**Age group**	18-35	30.56	25.87	32.53	28.18	29.49	28.29
	35-45	20.56	16.14	18.16	24.66	22.99	20.42
	45-55	32.44	21.48	32.30	25.28	19.38	25.92
	55-65	16.07	36.50	17.02	21.88	28.15	25.37
**Family**	No children	63.36	71.93	24.12	55.70	62.60	62.04
	Children ≤ 11	19.79	13.42	61.67	26.43	26.21	21.95
	Children > 11	16.86	14.65	14.21	17.87	11.18	16.01
**Education level**	Low	9.33	5.11	7.16	7.46	16.95	7.70
	Middle-Low	64.37	67.45	61.33	63.49	54.73	64.42
	High-Middle	18.24	9.51	14.60	12.08	16.62	13.18
	High	8.06	17.93	16.91	16.98	11.70	14.70
**Urban**	≤100.000	80.54	73.26	77.68	82.27	75.92	78.29
	>100.000	19.46	26.74	22.32	17.73	24.08	21.71
**Italy's Areas**	North-west	26.33	26.45	11.24	27.11	37.74	26.61
	North-East	20.89	17.00	44.38	16.75	23.64	19.49
	Centre	21.50	20.27	30.50	17.47	14.89	19.89
	South	20.70	23.37	2.11	28.15	21.55	23.15
	Islands	10.58	12.91	11.77	10.53	2.19	10.90
**Job**	Student	6.97	8.24	5.55	9.26	8.78	8.15
	Homely/Housewife	8.46	8.65	44.81	10.00	7.14	10.51
	Retired	4.07	7.36	0.00	1.90	1.02	4.12
	Unemployed or looking for a first job	7.85	8.14	1.24	5.77	9.40	7.10
	Manual worker	16.22	7.52	10.82	15.61	7.76	12.44
	Employe	35.20	37.86	21.88	36.60	41.01	36.27
	Self-employed	16.15	14.55	7.54	17.28	22.34	15.96
	Others	5.09	7.70	8.15	3.58	2.56	5.46
**Income**	<18.000€	18.05	29.87	4.94	17.46	24.91	21.67
	18.000-36.000€	52.73	45.44	87.79	50.35	37.24	50.25
	>36.000€	29.22	24.69	7.27	32.19	37.85	28.19
